# Mechanisms involved in PGE_2_-induced transactivation of the epidermal growth factor receptor in MH_1_C_1_ hepatocarcinoma cells

**DOI:** 10.1186/1756-9966-31-72

**Published:** 2012-09-11

**Authors:** Ingun Heiene Tveteraas, Kristin Meisdalen Müller, Monica Aasrum, John Ødegård, Olav Dajani, Tormod Guren, Dagny Sandnes, Thoralf Christoffersen

**Affiliations:** 1 Department of Pharmacology, Institute of Clinical Medicine, Faculty of Medicine, University of Oslo, P.O.Box 1057 Blindern, N-0316 Oslo, Norway; 2 Department of Oncology, Oslo University Hospital, Oslo, Norway

**Keywords:** EGF receptor, Prostaglandin receptors, Transactivation, Hepatocarcinoma cells, Hepatocytes

## Abstract

**Background:**

It is important to understand the mechanisms by which the cells integrate signals from different receptors. Several lines of evidence implicate epidermal growth factor (EGF) receptor (EGFR) in the pathophysiology of hepatocarcinomas. Data also suggest a role of prostaglandins in some of these tumours, through their receptors of the G protein-coupled receptor (GPCR) family. In this study we have investigated mechanisms of interaction between signalling from prostaglandin receptors and EGFR in hepatocarcinoma cells.

**Methods:**

The rat hepatocarcinoma cell line MH_1_C_1_ and normal rat hepatocytes in primary culture were stimulated with EGF or prostaglandin E_2_ (PGE_2_) and in some experiments also PGF_2α_. DNA synthesis was determined by incorporation of radiolabelled thymidine into DNA, phosphorylation of proteins in signalling pathways was assessed by Western blotting, mRNA expression of prostaglandin receptors was determined using qRT-PCR, accumulation of inositol phosphates was measured by incorporation of radiolabelled inositol, and cAMP was determined by radioimmunoassay.

**Results:**

In the MH_1_C_1_ hepatocarcinoma cells, stimulation with PGE_2_ or PGF_2α_ caused phosphorylation of the EGFR, Akt, and ERK, which could be blocked by the EGFR tyrosine kinase inhibitor gefitinib. This did not occur in primary hepatocytes. qRT-PCR revealed expression of EP1, EP4, and FP receptor mRNA in MH_1_C_1_ cells. PGE_2_ stimulated accumulation of inositol phosphates but not cAMP in these cells, suggesting signalling via PLCβ. While pretreatment with EP1 and EP4 receptor antagonists did not inhibit the effect of PGE_2_, pretreatment with an FP receptor antagonist blocked the phosphorylation of EGFR, Akt and ERK. Further studies suggested that the PGE_2_-induced signal was mediated via Ca^2+^ release and not PKC activation, and that it proceeded through Src and shedding of membrane-bound EGFR ligand precursors by proteinases of the ADAM family.

**Conclusion:**

The results indicate that in MH_1_C_1_ cells, unlike normal hepatocytes, PGE_2_ activates the MEK/ERK and PI3K/Akt pathways by transactivation of the EGFR, thus diversifying the GPCR-mediated signal. The data also suggest that the underlying mechanisms in these cells involve FP receptors, PLCβ, Ca^2+^, Src, and proteinase-mediated release of membrane-associated EGFR ligand(s).

## Background

Malignant cells are exposed to a variety of active agents, including hormones, peptide growth factors, cytokines, and many other locally acting substances such as prostaglandins, which together control or modulate the cellular functions. It is of interest to understand the mechanisms by which the cells integrate signals from different bioactive molecules via their receptors. A notable example is the interaction between pathways from G protein-coupled receptors (GPCRs) and receptor tyrosine kinases (RTKs). Studies in many cells have shown that signals from GPCRs may involve interaction with the epidermal growth factor receptor (EGFR), an ErbB family RTK [1-5]. EGFR, which serves important functions in normal cells [6,7], is involved in several malignancies [8,9], and is a target of novel antitumour therapies [10,11]. In studies including tumour cells from colon and pancreatic cancer, we have found that different mechanisms may be involved in the interaction of pathways from GPCRs and EGFR [12].

EGFR conveys strong mitogenic stimulation in normal hepatocytes [13-16], and several lines of evidence suggest a role of EGFR in hepatocarcinogenesis [17-20]. For example, overexpression of the EGFR agonist transforming growth factor alpha (TGFα) in mice causes hepatic hyperplasia and tumour formation [21,22], and EGFR is upregulated in a majority of human hepatocarcinomas [23]. Inhibition of the EGFR by antibodies or tyrosine kinase blockers can attenuate the growth of hepatocarcinoma cells in vitro [24,25], and are currently being tested in clinical trials in hepatocarcinomas [26].

Prostaglandins, acting through different receptors of the GPCR family, regulate many cellular functions [27]. In epithelial cells, prostaglandins often enhance proliferation and survival, and several lines of evidence implicate them in oncogenesis [28]. In many tumours, cyclooxygenases (COX-1 and COX-2), which catalyze the rate-limiting step in prostaglandin synthesis, are overexpressed, and the levels of prostaglandins, notably prostaglandin E_2_ (PGE_2_), are elevated [28-31]. In hepatocytes, PGE_2_ and other prostaglandins enhance DNA synthesis [15,32-34], and COX-2 is overexpressed in many hepatocarcinomas [35,36].

In the study presented here we examined the Morris hepatocarcinoma cell line MH_1_C_1_, which was chosen due to its responsiveness to both EGF and the prostaglandins PGE_2_ and PGF_2α_, and investigated the interaction between the pathways mediated by prostaglandin receptors and EGFR. We previously observed that while there was no evidence of transactivation of EGFR induced by prostaglandins or other GPCR agonists in hepatocytes, PGE_2_ induced phosphorylation of the EGFR in the MH_1_C_1_ cells [37,38]. We have now investigated further the signalling mechanisms involved in this effect.

## Methods

### Chemicals

Dulbecco’s Modified Eagle's Medium, Dulbecco’s phosphate-buffered saline, William’s Medium E, glutamine, and Pen-Strep (10.000 U/ml) were from Lonza(Verviers, Belgium). HEPES was from Gibco (Grand Island, NY). Dexamethasone, insulin, bovine serum albumin, collagen (type I, rat tail), prostaglandin F_2α_ (Tris salt) and epidermal growth factor (EGF) were obtained from Sigma-Aldrich (St.Louis, MO). GF109203X ([2-[1-(3-dimetylaminopropyl)-1 *H*-indol-3-yl]-male-imide]) and GM6001/Galardin (N-[(2R)-2 (hydroxamidocarbonylmethyl)-4-methylpentanoyl]-L-tryptophan methylamide) were from Calbiochem (San Diego, CA). Gefitinib was a gift from AstraZeneca (Cheshire, UK). [6-^3^ H]thymidine (20–30 Ci/mmol) and m*yo*-[2-^3^ H]inositol (15.0 Ci/mmol) were from PerkinElmer (Boston, MA). AL8810 (9α,15R-dihydroxy-11β-fluoro-15-(2,3-dihydro-1 H-inden-2-yl)-16,17,18,19,20-pentanor-prosta-5Z,13E-dien-1-oic acid),L161982 (N-[[4'-[[3-butyl-1,5-dihydro-5-oxo-1-[2-(trifluoromethyl)phenyl]-4 H-1,2,4-triazol-4-yl]methyl][1,1'-biphenyl]-2-yl]sulfonyl]-3-methyl-2-thiophenecarboxamide), (+)fluprostenol, and prostaglandin E_2_ (PGE_2_) were from Cayman Chemical (Ann Arbor, MI). SC51322 (8-chloro-2-[3-[(2-furanylmethyl)thio]-1-oxopropyl]hydrazide, dibenz[b,f][1,4]oxazepine-10(11 H)-carboxylic acid) was obtained from BIOMOL Research Laboratories (Plymouth Meeting, PA). The Src inhibitor CGP77675 was a gift from Novartis Pharma AG (Basel, Switzerland). All other chemicals were of analytical quality.

Antibodies against phosphorylated Akt^Ser473^, total Akt, dually phosphorylated ERK^Thr202/Tyr204^, GAPDH and phospho-Shc^Tyr239/240^ were obtained from Cell Signaling Technology (Boston, MA). Antibody against phospho-EGF receptor^Tyr1173^ was obtained from Invitrogen. Anti-ERK antibody was from Upstate/Millipore (Billerica, MA). Secondary antibodies were purchased from Bio-Rad Laboratories (Hercules, CA) and Licor Biosciences (Lincoln, NE).

### Cells and culturing

The rat hepatocarcinoma cell line MH_1_C_1_, derived from a Morris hepatoma [39], was obtained from ATCC (Manassas, VA). The cells were seeded onto Costar plastic flasks and cultured in Dulbecco’s Modified Eagle’s medium. The Medium was supplemented with horse serum (10%), glutamine (2 mM), and 100 U/ml Pen-Strep. The cultures were kept in a humidified 5% CO_2_ incubator at 37°C. Cells were seeded onto culture wells at a density of 40 000–50 000 cells per cm^2^. After 24 hours, the medium was changed and the cells were cultured under serum-free conditions 24 h prior to stimulation.

Hepatocytes were isolated from male Wistar rats as previously described [40]. The hepatocytes were seeded onto Costar plastic culture wells at a density of 15 000–20 000 per cm^2^. The culture medium was a serum-free 1:1 combination of William’s Medium E and Dulbecco’s Modified Eagle’s Medium. The medium was supplemented with 100 U/ml Pen-Strep, collagen (3 μg/ml), insulin (100 nM) and dexamethasone (25 nM).

### Immunoblotting

Aliquots containing ~30000 MH_1_C_1_ cells or hepatocytes (total cell lysate prepared in Laemmli or RIPA buffer) were electrophoresed on 6–12% (w/v) polyacrylamide gels (acrylamide: N’N’-bis-methylene acrylamide 30:1). This was followed by protein electrotransfer to nitrocellulose membranes and immunoblotting with antibodies against proteins as described in the figures. Usually the same membrane was stripped and reincubated with different antibodies, and then one single loading control was used as the final incubation. Immunoreactive bands were visualized with enhanced chemiluminescence using LumiGLO (KPL Protein research Products, Gaithersburg, MD) or by infrared imaging using Odyssey Infrared Imaging System, supplied by Licor Biosciences (Lincoln, NE).

### RNA isolation and cDNA synthesis

RNA from MH_1_C_1_ cells was isolated with Qiagen RNeasy kit according to the manufacturer’s instructions, and was treated with DNAse. The integrity of RNA was evaluated by ethidium bromide agarose gel electrophoresis, and the quantity and purity was measured spectrophotometrically (OD 260/280). cDNA was synthesized from 1.0 μg RNA with Superscript® III reverse transcriptase (Invitrogen) according to manufacturer’s protocol. Reactions without reverse transcriptase were run in parallel to control for contamination with chromosomal DNA. Standard curves with RNA ranging from 0.25 to 2.0 μg of total RNA were made to control for the reverse transcription and PCR quantification.

### *Quantitative real-time PCR* (qRT-PCR)

The cDNA was analyzed in triplicate by real time quantitative PCR on an ABI Prism 7900 HT Sequence detector (Applied Biosystems) with the following cycling parameters: 50°C for 2 min, 95°C for 10 min and 40 cycles of 15 s at 95°C and 60 s at 60°C, followed by melting point analysis when using SYBR green. Raw data were collected and analyzed in the Sequence Detector Software (SDS ver. 2.2, Applied Biosystems), and cycle of threshold value (Ct) was calculated from each amplification plot. Standard curves (Ct value versus log initial RNA concentration) were used to calculate the relative input amount of RNA for each sample based on the Ct value [41]. Satisfactory and comparable amplification efficiency was verified by the slopes of standard curves. Primers were designed using Primer Express® software v2.1 (ABI Prism, Applied Biosystems), and were validated by the production of single products of expected size on agarose gels, as well as uniformity of melting temperature, which was routinely performed. Prostaglandin receptor cDNA was detected with SYBR Green methodology and the following primers: EP1: forward 5’-CCT GCT GGT ATT GGT GGT GTT-3’ and reverse 5’-GGG GTA GGA GGC GAA GAA GTT-3’; EP2: forward 5’-GCT CCC TGC CTT TCA CAA TCT-3’ and reverse 5’-GGA CTG GTG GTC TAA GGA TGA CA-3’; EP3: forward 5’-GGT CGC CGC TAT TGA TAA TGA T-3’ and reverse 5’-CAG GCG AAC GGC GAT TAG-3‘; EP4: forward 5’-CTC GTG GTG CGA GTG TTC AT-3’ and reverse 5’-TGT AGA TCC AAG GGT CCA GGA T-3’; FP: forward 5’-GTC ATT CAG CTC CTG GCC ATA-3’ and reverse 5’-AGC GTC GTC TCA CAG GTC ACT-3’. GAPDH cDNA was quantified using the dual hybridization probe Double Dye oligonucleotide 5’ labelled with the fluorescent dye Yakima yellow and quenched with Dark Quencher, 5’-CTC ATG ACC ACA GTC CAT GCC ATC ACT-3’ and the following primers: forward 5’-CCA AGG TCA TCC ATG ACA ACT T-3’ and reverse 5’-AGG GGC CAT CCA CAG TCT T-3’. Results were normalized to GADPH.

### Accumulation of inositol phosphates and **cAMP**

^3^ H]inositol, 5 μCi/well was added simultaneously with the serum-free medium. 30 minutes before agonist stimulation for 30 minutes in serum-starved cells, medium was removed and replaced with Krebs-Ringer-Hepes buffer pH 7.4, containing 10 mM glucose and 15 mM LiCl. MH_1_C_1_ cells were stimulated with PGE_2_, fluprostenol or isoproterenol as indicated, and the reaction was stopped by removing buffer and adding 1 ml ice-cold 0.4 M perchloric acid. Samples were harvested and neutralized with 1.5 M KOH, 60 mM EDTA and 60 mM Hepes, in the presence of Universal indicator. The neutralized supernatants were applied on columns containing 1 ml Dowex AG 1-X8 resin. The columns were washed with 20 ml distilled water and 10 ml 5 mM sodium tetraborate/60 mM ammonium formate, and inositol phosphates were eluted with 10 ml 1 M ammonium formate/0.1 M formic acid. cAMP was determined by radioimmunoassay as previously described [42].

### Measurement of DNA synthesis

MH_1_C_1_ cells were seeded onto culture wells, and after 24 hours, the medium was changed and the cells were cultured under serum-free conditions. 24 h after change to serum-free medium, cells were treated with various concentrations of gefitinib and harvested at 48 hours, after three hours of pulsing with ^3^ H]thymidine. DNA synthesis was measured as the amount of radioactivity incorporated into DNA as previously described [34].

## Results

In preliminary experiments we investigated the effect of PGE_2_ in the rat hepatocarcinoma cell lines MH_1_C_1_, McA7777, and M4IIE, and the human hepatocarcinoma cell line HepG2. Although some of these cell lines had strong responses to EGF (data not shown), the MH_1_C_1_ were the only cells showing consistent responses to both EGF and prostaglandins, and we therefore used these cells in further experiments.

### Transactivation of EGFR induced by PGE_2_ and PGF_2α_ in MH_1_C_1_ cells

**Figure 1:**
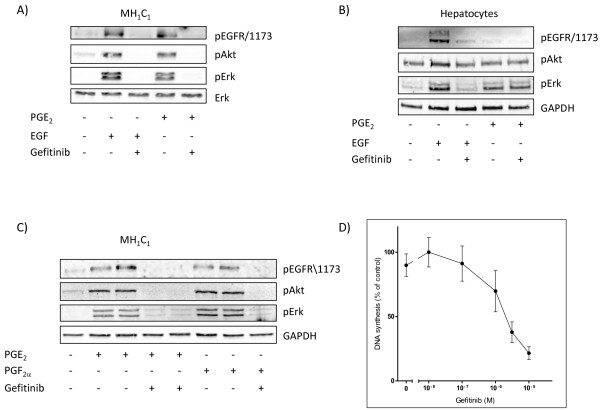
**Effects of the EGFR inhibitor gefitinib on phosphorylation of signalling proteins and DNA synthesis. A**) MH_1_C_1_ cells were treated with gefitinib (1 μM) for 30 min before stimulation with EGF (10 nM) or PGE_2_ (100 μM) for 5 min. **B**) Hepatocytes were treated with gefitinib (1 μM) for 30 min before stimulation with EGF (10 nM) or PGE_2_ (100 μM) for 5 min. **C**) Gefitinib (1 μM) was added 30 min prior to stimulation with either PGE_2_ (100 μM) or PGF_2α_ (100 μM) for 5 min. Cells were harvested and subjected to SDS-PAGE followed by immunoblotting with antibodies and detection with enhanced chemiluminescence as described in Materials and Methods. All blots are representative of at least 3 independent experiments. **D**) Effect of gefitinib on DNA synthesis in MH_1_C_1_ cells. Increasing concentrations of gefitinib were added to serum-starved MH_1_C_1_ cells. [^3^ H]thymidine was added, and DNA synthesis was assessed as described under Materials and Methods. The results are presented as percent of control ± S.E.M of four independent experiments.

We previously observed that in the MH_1_C_1_ cells, unlike normal hepatocytes, PGE_2_ induced phosphorylation of the EGFR and activated ERK by a mechanism that was sensitive to EGFR inhibition [37]. Further investigation (Figure 1), showed that in addition to inducing phosphorylation of EGFR and ERK, PGE_2_ treatment also led to phosphorylation of Akt. All these effects were inhibited by gefitinib (1 μM) (Figure 1A), providing further support for a transactivation of EGFR in the MH_1_C_1_ cells. In contrast, the effects of PGE_2_ on ERK and Akt in hepatocytes were not dependent on the EGFR, since they were not inhibited by gefitinib (Figure 1B). We also observed that in the MH_1_C_1_ cells, the phosphorylation of the EGFR was somewhat slower after stimulation with PGE_2_ than with EGF (data not shown), suggesting an indirect mechanism consistent with PGE_2_-induced transactivation. As shown in Figure 1C, PGF_2α_ also induced a gefitinib-sensitive phosphorylation of EGFR, Akt and ERK in these cells.

Figure 1D shows that the EGFR tyrosine kinase blocker gefitinib dose-dependently inhibited DNA synthesis in MH_1_C_1_, indicating that EGFR is involved in the growth in these cells. Most likely there is an autocrine release of EGFR agonist(s) in these long-term experiments (48 h culturing). This has not been explored further in the present study, as the experiments below focus on early receptor-mediated mechanisms.

### Prostaglandin receptors and involvement of PLCβ

**Figure 2:**
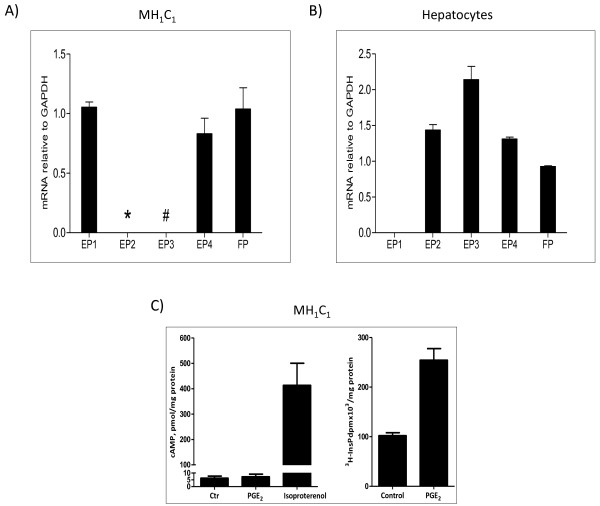
**Prostaglandin receptors and cAMP and PLCβ responses. A**) and **B**) Expression of prostaglandin receptor mRNA in MH_1_C_1_ cells (data from three experiments, measured in triplicate) and hepatocytes (data from one experiment measured in triplicate). Quantitative RT-PCR of EP1, EP2, EP3, EP4 and FP normalized to GADPH. RNA was isolated as described in Materials and Methods. * not detected # low levels-not quantifiable. **C**) Left: Accumulation of cAMP in MH_1_C_1_ cells after stimulation with either PGE_2_ (100 μM) or isoproterenol (10 μM) in the presence of 0.5 mM IBMX. cAMP was measured after 3 minutes. Right: Accumulation of inositol phosphates in MH_1_C_1_ cells after stimulation with PGE_2_ (100 μM) for 30 minutes in the presence of 15 mM LiCl. The data shown are mean ± S.E.M of three independent experiments.

We next investigated which prostaglandin receptors are expressed in the MH_1_C_1_ cells. qRT-PCR analysis revealed mRNA expression of EP1, EP4, and FP subtypes of prostaglandin receptors, whereas only traces of EP3 receptor mRNA were present and no EP2 expression was detected (Figure 2A). The hepatocytes expressed EP2, EP3, EP4, and FP (Figure 2B).

The available evidence indicates that the EP4 receptors are coupled to Gs proteins and adenylyl cyclase activity and thereby cAMP elevation, and that FP receptors couple to Gq proteins which mediate activation of phospholipase C-β (PLCβ) leading to formation of inositol trisphosphate (InsP_3_) and diacylglycerol (DAG) [27,43]. The G proteins and signalling mechanisms stimulated by the EP1 receptors are not fully clarified [43,44]. PGE_2_ has high affinity for EP1 and EP4 receptors, and while the FP receptor has the highest affinity for PGF_2α_, PGE_2_ also binds to this receptor [27]. In the MH_1_C_1_ cells no cAMP response to PGE_2_ could be detected, although the cells had a functional adenylyl cyclase, as shown by their marked cAMP elevation in response to the β-adrenergic agonist isoproterenol (Figure 2C left). In contrast, PGE_2_ stimulated accumulation of inositol phosphates (Figure 2C right). Thus, it is likely that PGE_2_ induces signalling through PLCβ activation in these cells.

**Figure 3:**
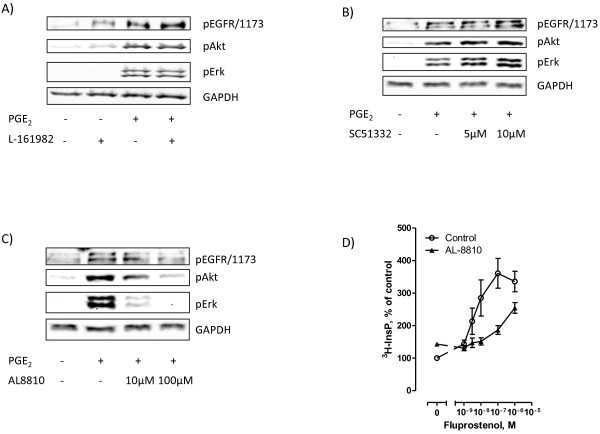
**Effect of different prostaglandin receptor inhibitors in MH**_**1**_**C**_**1 **_**cells. A**) The EP4 inhibitor L-161982 (10 μM) was added 30 min prior to stimulation with PGE_2_ (100 μM) for 5 min. **B**) The EP1 inhibitor SC51322 (5 or 10 μM) was added 30 minutes prior to stimulation with PGE_2_ (100 μM) for 5 min. **C**) The FP inhibitor AL8810 (10 or 100 μM) was added 30 minutes prior to stimulation with PGE_2_ (100 μM) for 5 min. All blots are representative of three independent experiments. **D**) Effect of AL8810 (100 μM) on accumulation of inositol phosphates after stimulation with increasing concentrations of fluprostenol for 30 minutes in the presence of 15 mM LiCl. The data shown are mean ± S.E.M of four independent experiments.

To investigate which receptors are involved in the EGFR transactivation by PGE_2_, we studied the effect of pretreating the cells with selective inhibitors of different prostaglandin receptors. The results suggested that EP4 did not mediate this transactivation since the EP4 receptor antagonist L161982 did not inhibit the effect of PGE_2_ on the phosphorylation of EGFR, Akt, or ERK (Figure 3A), consistent with the lack of PGE_2_-induced cAMP response in these cells (Figure 2C). We then examined the roles of EP1 and FP receptors. Pretreatment of the cells with 10 μM of the EP1 receptor antagonist SC51322 did not affect PGE_2_-induced phosphorylation of EGFR, Akt, or ERK (Figure 3B). In contrast, the FP receptor antagonist AL8810 at 10 μM significantly inhibited the effect of PGE_2_ on the phosphorylation of ERK, while 100 μM inhibited phosphorylation of EGFR and Akt and blocked the effects on ERK almost completely (Figure 3C). These concentrations of AL8810 were not toxic to the cells. Although AL8810 is a less potent antagonist than L161982 or SC51322 [27,45,46], it was the only antagonist that had effect at 10 μM. It was previously shown that at 10 μM, AL8810 did not inhibit functional responses through other prostaglandin receptors, suggesting that it is a selective antagonist at the FP receptor [45]. Further support for a functional role of FP receptors in these cells was obtained in the results given in Figure 3D, demonstrating that AL8810 inhibited the inositol phosphate accumulation induced by the FP receptor agonist fluprostenol. Taken together, these results suggest that the PGE_2_-induced transactivation of EGFR in MH_1_C_1_ hepatoma cells is mediated primarily by FP receptors and signalling via Gq and PLCβ.

### Evidence of a role for Ca^2+^, but not PKC, in the PGE_2_-induced transactivation of EGFR

**Figure 4:**
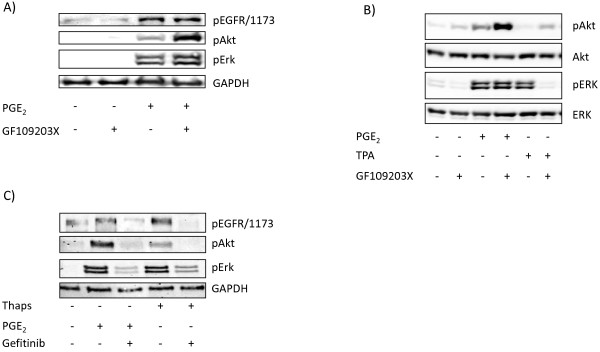
**Role of Ca**^**2+ **^**and PKC in responses to PGE**_**2 **_**in MH**_**1**_**C**_**1 **_**cells. A**) MH_1_C_1 _cells were pretreated for 30 min with the PKC inhibitor GF109203X (3.5 μM) before stimulation with PGE_2_ (100 μM) for 5 min. **B**) MH_1_C_1_ cells were pretreated for 30 min with the PKC inhibitor GF109203X (3.5 μM) before stimulation with PGE_2_ (100 μM) or TPA (1 μM) for 5 min. **C**) MH_1_C_1_ cells were treated with gefitinib (1µM) for 30 min before stimulation with either PGE_2_ (100 μM) or thapsigargin (1 μM) for 5 min. Cells were then harvested and subjected to immunoblot analysis as described in Materials and Methods. Representative blots of at least three experiments.

We next tried to determine which pathways downstream of PLCβ are mediating the PGE_2_-induced transactivation of EGFR. InsP_3_ and DAG stimulate cytosolic Ca^2+^ release and protein kinase C (PKC) activity, respectively. Pretreatment of the cells with the PKC inhibitor GF109203X did not prevent the effects of PGE_2_ on the phosphorylation of the EGFR, ERK, or Akt in the MH_1_C_1_ cells (Figure 4A). Furthermore, the data in Figure 4B, comparing PGE_2_ and the direct PKC activator tetradecanoylphorbol acetate (TPA), showed that TPA did not mimic the effect of PGE_2_ on Akt, and its stimulation of ERK, unlike the effect of PGE_2_, was blocked by GF109203X. Interestingly, pretreatment of the cells with GF109203X consistently increased basal and PGE_2_-induced Akt phosphorylation in the cells. This might result from a reduced feedback inhibition by PKC [47]. In contrast to TPA, thapsigargin, which increases the intracellular Ca^2+^ level by inhibiting the ‘sarco/endoplasmic reticulum Ca^2+^-ATPase’ (SERCA) pump [48], induced gefitinib-sensitive phosphorylation of EGFR, ERK, and Akt (Figure 4C). Taken together, these data suggest that Ca^2+^ rather than PKC mediates the PGE_2_-induced transactivation of the EGFR in these cells.

### Role of Src and metalloproteinases in the transactivation of the EGFR

**Figure 5:**
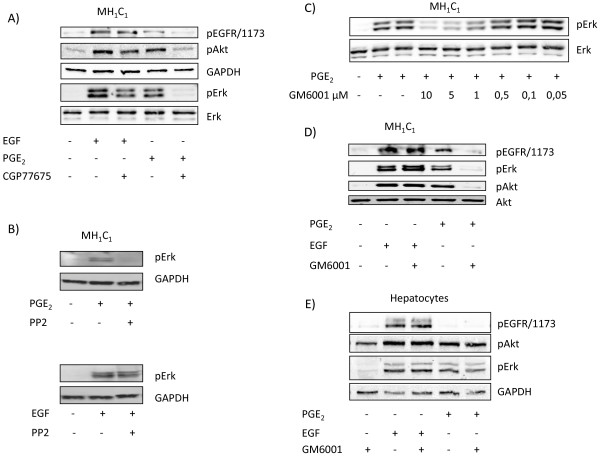
**Effect of Src and MMP inhibitors on phosphorylation of EGFR and downstream targets. A**) MH_1_C_1_ cells were pretreated for 90 min with the Src inhibitor CGP 77675 (10 μM). Cells were then stimulated with either PGE_2_ (100 μM) or EGF (10 nM) for 5 min before they were harvested and immunoblotting performed as described in Materials and Methods. Representative blots of at least three experiments. **B**) MH_1_C_1_ cells were pretreated for 30 min with the Src inhibitor PP2 (10 μM). Cells were then stimulated with either PGE_2_ (100 μM) or EGF (10 nM) for 5 min before they were harvested and immunoblotting performed as described in Materials and Methods. Representative blots of two experiments. **C**) MH_1_C_1_ cells were pretreated for 30 min with increasing concentrations of the metalloproteinase inhibitor GM6001. Cells were then stimulated with PGE_2_ (100 μM) for 5 min before they were harvested and immunoblotting performed as described in Materials and Methods. Representative blots of three experiments **D**) MH_1_C_1_ cells were pretreated for 30 min with the metalloproteinase inhibitor GM6001 (10 μM). Cells were then stimulated with either PGE_2_ (100 μM) or EGF (10 nM) for five minutes before they were harvested and immunoblotting performed as described in Materials and Methods. Representative blots of at least three experiments **E**) Same experiment as in **D**) performed in hepatocytes. Representative blots of at least three experiments.

To further elucidate mechanisms involved in transactivation of the EGFR, we investigated the effects of Src inhibitors. As shown in Figure 5A, pretreatment of the cells with the Src inhibitor CGP77675 almost completely abolished the PGE_2_-induced phosphorylation of EGFR and the activation of ERK and Akt, but, in contrast, had little or no effect on the phosphorylation of these proteins elicited by EGF. The Src inhibitor PP2 similarly prevented the phosphorylation of ERK in response to PGE_2_, while the response to EGF was not significantly affected (Figure 5B). These results suggest an involvement of a Src family kinase in the PGE_2_-induced transactivation of EGFR in MH_1_C_1_ cells.

Previous evidence has implicated proteinases of the ‘a-disintegrin-and-metalloproteinase’ (ADAM) family in EGFR transactivation by GPCRs in various cells [2,49,50]. To test the role of ADAMs in the PGE_2_-induced EGFR transactivation in MH_1_C_1_, we pretreated the cells with GM6001, which is a broad-spectrum metalloproteinase inhibitor [50]. This pretreatment resulted in complete inhibition of PGE_2_-induced phosphorylation of EGFR, ERK, and Akt, while the EGF-induced phosphorylation of these proteins was not affected (Fig 5C and D), indicating that the transactivation is dependent on mechanisms involving ADAM-mediated release of EGFR ligand(s). We also examined the effect of this inhibitor in the primary cultures of rat hepatocytes, and found neither inhibition of PGE_2_-induced phosphorylation of ERK and Akt in these cells nor any effect on EGF-induced phosphorylation of EGFR, ERK and Akt (Figure 5E).

## Discussion

**Figure 6:**
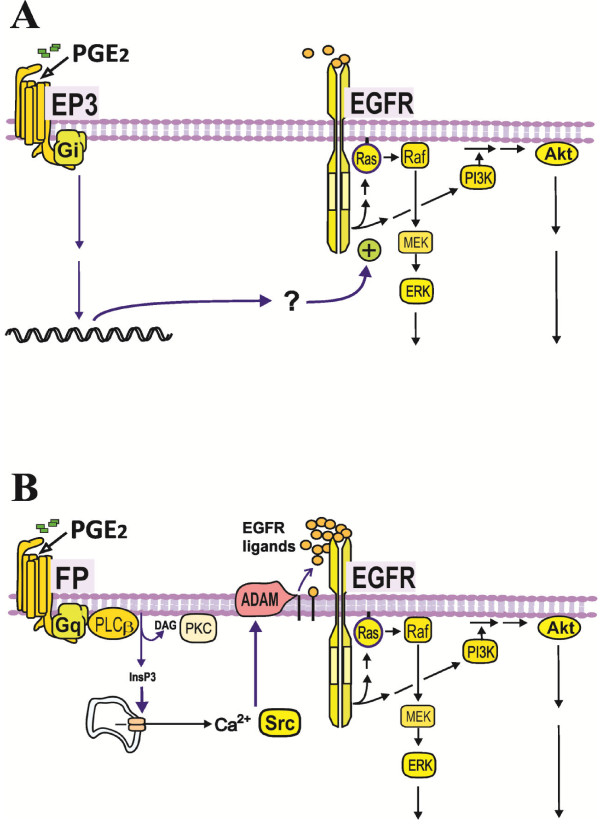
**Mechanisms by which PGE**_**2 **_**interacts with EGFR-mediated signalling in hepatocytes and MH**_**1**_**C**_**1 **_**hepatocarcinoma cells. A**) In normal rat hepatocytes, PGE_2_ does not elicit transactivation of EGFR, but induces upregulation of the effectiveness in Ras/ERK and PI3K/Akt pathways downstream of EGFR, leading to an enhanced mitogenic response to EGF family growth factors [37,38,51]. Although not fully clarified, previous studies have indicated that this effect of PGE_2_ is mediated primarily through EP3 receptors and Gi proteins, requires several hours to develop, and is most likely a result of altered gene expression [34,37,38,51,52]. **B**) In MH_1_C_1_ rat hepatocarcinoma cells, PGE_2_ transactivates EGFR and thereby activates the Ras/ERK and PI3K/Akt signalling pathways. The results of the present study suggest that this effect is exerted via FP receptors, Gq proteins, PLCβ, intracellular Ca^2+^ (but not PKC), Src, and ADAM-mediated release of EGFR ligands.

We have shown that in the MH_1_C_1_ hepatocarcinoma cells stimulation with PGE_2_ or PGF_2α_ causes phosphorylation of the EGFR and an EGFR-dependent phosphorylation of ERK and Akt, indicating that these prostaglandins induced transactivation of EGFR. Further study of the PGE_2_ effect suggested that the transactivation was mediated by the Gq-coupled FP receptor and activation of PLCβ with downstream signalling by Ca^2+^ release, Src, and ADAM-mediated shedding of membrane-bound EGFR ligand precursors. In contrast, in primary hepatocytes, PGE_2_ did not phosphorylate the EGFR, and gefitinib did not prevent phosphorylation of Akt or ERK after PGE_2_-stimulation, which lends further support to our previous data suggesting that GPCR agonists do not transactivate the EGFR in normal rat hepatocytes, but rather signal via mechanisms that synergistically enhance the effects of EGF [34,37,38,51,52] (Figure 6).

Different receptors and pathways may be involved in mitogenic and tumour-promoting effects of prostaglandins [28]. qRT-PCR analysis showed that the prostaglandin receptors expressed in these cells are EP1, EP4, and FP. No significant increase in cAMP accumulation was detected, in accordance with previous results [53], suggesting either that the EP4 protein levels are low, or that these receptors are functionally uncoupled from adenylyl cyclase. In contrast, PGE_2_ stimulated accumulation of inositol phosphates. Pretreatment with the EP4 antagonist L161982 or the EP1 antagonist SC51322, had no effect on the PGE_2_-induced phosphorylation of EGFR, ERK, or Akt, while the phosphorylation of these proteins were markedly inhibited by the FP antagonist AL8810. PGF_2α_, which binds to FP receptors with high affinity, mimicked the effects of PGE_2_. Together, these results suggest that in contrast to the normal rat hepatocytes, where the effect of PGE_2_ seems to be mediated primarily through the EP3 receptor [37,52,54], the MH_1_C_1_ cells, which do not express EP3 receptors, respond to PGE_2_ through FP receptors, Gq, and PLCβ. It is of interest that expression of EP3 receptors has been found to be suppressed or absent in colon cancer in vivo and in vitro, as compared to normal mucosa [55].

PLCβ can regulate cellular functions via two distinct pathways, involving DAG-mediated activation of PKC and InsP_3_-induced release and elevation of cytosolic Ca^2+^, respectively. Our findings suggest that in the MH_1_C_1_ cells, the effect of PGE_2_ was mediated through Ca^2+^, since it was not mimicked by TPA and not inhibited by a PKC blocker, while thapsigargin, which elevates intracellular Ca^2+^, mimicked the PGE_2_ effect, inducing a gefitinib-sensitive phosphorylation of EGFR.

In other cells, both ligand-dependent and ligand-independent mechanisms have been found to mediate EGFR transactivation [5]. Ligand-dependent mechanisms involve the release of EGFR agonists by cleavage and shedding of membrane-associated precursors by proteinases of the ADAM family [2,49]. Ligand-independent mechanisms have been suggested to involve intracellular molecules including Src family kinases and Pyk2 [1,3,56,57]. Han et al. reported that in Hep3B cells, PGE_2_ induced phosphorylation of the EGFR through EP1 receptors and an intracellular mechanism involving Src [57]. Itabashi et al. demonstrated that in some hepatocarcinoma cell lines EGFR transactivation triggered by angiotensin II stimulation was mediated through release of EGFR ligand by members of the ADAM family [58]. In the MH_1_C_1_ cells, we observed that Src inhibitors abolished PGE_2_-stimulated phosphorylation of the EGFR, ERK, and Akt, but in contrast, only slightly affected the response to EGF, suggesting a role of Src in the transactivation in these cells. We also found evidence for the involvement of ligand shedding in the transactivation of EGFR after PGE_2_ stimulation, since pretreatment of the cells with the metalloproteinase inhibitor GM6001 almost completely prevented PGE_2_-induced, but not EGF-induced, phosphorylation of EGFR, Akt and ERK. GM6001 did not affect the effects of PGE_2_ in the normal hepatocytes. The lack of transactivation in response to PGE_2_ in these cells could be due to the low expression of metalloproteinases in hepatocytes as compared to hepatocarcinoma cells [59]. These results indicate that in the MH_1_C_1_ cells Src is involved in activating ADAMs rather than directly stimulating the EGFR, thus combining the two mechanisms. The involvement of both Src and ADAMs has been reported in normal gastrointestinal epithelial and colon cancer cell lines [60].

Several signalling pathways seem to be important in hepatocarcinomas [19], and there is evidence that both EGFR-mediated mechanisms and the COX/prostaglandin system may be involved in the pathobiology of these tumours [17,18,20,35,36]. The results of the present study suggest a functional interaction between the EGFR and the prostaglandins. It has been proposed that transactivation can explain the mitogenic effect of GPCR ligands in some cell systems [61] and that it represents a means of diversifying signalling in the cells, by linking the input from a large number of ligands stimulating GPCRs to the pleiotypic and potentially tumorigenic effects of the EGFR [62]. However, there seems to be great variation between cell types with respect to the different pathways involved in the signalling. We have recently shown that while neurotensin, a GPCR agonist, activates ERK and Akt in an EGFR-independent way in pancreatic cancer Panc-1 cells, as also found by others [63], and activates ERK and Akt via EGFR transactivation in the colon cancer cell line HT 29, neurotensin uses both EGFR-dependent and -independent pathways in the colon cancer cell line HCT 116 [12].

In the present study we have shown that PGE_2_ has different ways of stimulating the cells, acting by FP-mediated EGFR transactivation in the hepatocarcinoma cells, whereas the effect is mediated mainly via EP3 receptors without any involvement of the EGFR in the hepatocytes [37,52]. This is further evidence of the diversity of intracellular cross-talk and underscores the importance of investigating such mechanisms in order to better understand the signalling in cancer cells.

## Conclusion

The results indicate that in MH_1_C_1_ cells, unlike normal hepatocytes, PGE_2_ activates the MEK/ERK and PI3K/Akt pathways by transactivation of the EGFR, thus diversifying the GPCR-mediated signal. The data also suggest that the underlying mechanisms in these cells involve FP receptors, PLCβ, Ca^2+^, Src, and proteinase-mediated release of membrane-associated EGFR ligand(s).

## Abbreviations

ADAM: A disintegrin and metalloproteinase; DAG: Diacylglycerol; EGF: Epidermal growth factor; EGFR: Epidermal growth factor receptor; ERK: Extracellular regulated kinase; EP: E prostaglandin; FP: F prostaglandin; GPCR: G protein-coupled receptor; InsP_3_: Inositol trisphosphate; PG: Prostaglandin; PGE_2_: Prostaglandin E_2_; PGF_2α_: Prostaglandin F_2α_; PLCβ: Phospholipase C-β; PKC: Protein kinase C; RTK: Receptor tyrosine kinase; TPA: Tetradecanoylphorbol acetate; SERCA: Sarco/endoplasmic reticulum Ca^2+^-ATPase.

## Competing interests

The authors declare that they have no competing interests.

## Authors’ contributions

IHT participated in the design of the study, carried out immunoblotting experiments and drafted the manuscript. KMM carried out immunoblotting experiments, inositol phosphate experiments and helped revise the manuscript. MA helped revise the manuscript. JØ carried out qRT-PCR experiment and helped revise the manuscript. OD conceived of the study, carried out DNA synthesis and helped revise the manuscript. TG conceived of the study and helped revise the manuscript. DS conceived of the study, participated in the design of the study, carried out cAMP and inositol phosphate experiments and helped revise the manuscript. TC conceived of the study, participated in the design of the study and helped revise the manuscript. All authors read and approved of the final manuscript.
